# Technologies and Services to Support Care: Caregivers’ Experiences and Perspectives

**DOI:** 10.1093/geroni/igab046.699

**Published:** 2021-12-17

**Authors:** Chaiwoo Lee

**Affiliations:** Massachusetts Institute of Technology, Cambridge, Massachusetts, United States

## Abstract

Technologies developed to make life easier for the general population – including smart home products, internet-enabled services, communication platforms, and health management systems – also have the potential to assist individuals who provide care to loved ones. While caregivers may be eager users of technology to support their responsibilities, some technologies remain untapped resources. An in-depth survey conducted with the MIT AgeLab Caregiver Panel around attitudes toward and use of technology for themselves and for caregiving showed that while caregivers use a wide range of technologies for themselves, their use for caregiving is limited. However, while caregivers did not universally use technologies or services to support the care they provided, those who did so generally reported positive feelings about their use. This presentation will report on technology experiences – including perceived usefulness, ease of use and integration, impacts, and overall satisfaction – among caregivers of various characteristics and conditions.

